# Formation-Damage Mechanism and Gel-Breaker-Free Drill-In Fluid for Carbonate Reservoir

**DOI:** 10.3390/gels8090565

**Published:** 2022-09-06

**Authors:** Qingchao Fang, Xin Zhao, Hao Sun, Zhiwei Wang, Zhengsong Qiu, Kai Shan, Xiaoxia Ren

**Affiliations:** 1 Shandong Key Laboratory of Oilfield Chemistry, China University of Petroleum (East China), Qingdao 266580, China; 2 School of Petroleum Engineering, China University of Petroleum (East China), Qingdao 266580, China; 3 Research Institute of Petroleum Engineering and Technology, Sinopec Shengli Oilfield Company, Dongying 257000, China; 4 School of Science, Qingdao University of Technology, Qingdao 266525, China

**Keywords:** carbonate reservoir, formation damage, drill-in fluid, improved ideal filling for temporary plugging, gel-breaker free

## Abstract

Abundant oil and gas reserves have been proved in carbonates, but formation damage affects their production. In this study, the characteristics and formation-damage mechanism of the carbonate reservoir formation of the MS Oilfield in the Middle East were analyzed—utilizing X-ray diffraction, a scanning electron microscope, slice identification, and mercury intrusion—and technical measures for preventing formation damage were proposed. An ‘improved ideal filling for temporary plugging’ theory was introduced, to design the particle size distribution of acid-soluble temporary plugging agents; a water-based drill-in fluid, which did not require gel-breaker treatment, was formed, and the properties of the drill-in fluid were tested. The results showed that the overall porosity and permeability of the carbonate reservoir formation were low, and that there was a potential for water-blocking damage. There were micro-fractures with a width of 80–120 μm in the formation, which provided channels for drill-in fluid invasion. The average content of dolomite is 90.25%, and precipitation may occur under alkaline conditions. The polymeric drill-in fluid had good rheological and filtration properties, and the removal rate of the filter cake reached 78.1% in the chelating acid completion fluid without using gel breakers. In the permeability plugging test, the drill-in fluid formed a tight plugging zone on the surface of the ceramic disc with a pore size up to 120 μm, and mitigated the fluid loss. In core flow tests, the drill-in fluid also effectively plugged the formation core samples by forming a thin plugging layer, which could be removed by the chelating acid completion fluid, indicated by return permeability higher than 80%. The results indicated that the drill-in fluid could mitigate formation damage without the treatment of gel breakers, thus improving the operating efficiency and safety.

## 1. Introduction

A large number of oil and gas reserves are in carbonate formations. It is reported that the oil and gas production of carbonate reservoirs accounts for about 60% of the total oil and gas production in the world [1]. At present, most developed carbonate reservoirs are fractured formations with poor physical properties, uncertain fracture aperture, and strong heterogeneity [2,3,4]. The reservoir formation is easily damaged in drilling and completion operations, and the damage is not easily repaired, resulting in the decline of production [5,6,7,8]. Therefore, it is necessary to use low-damage drilling fluid (reservoir drill-in fluid) to minimize formation damage during drilling and completion operations [9,10,11,12].

Oil and gas resources in the Middle East are extremely rich, and the main target reservoir formation of some oil fields is subsalt carbonate formations. The MS Oilfield is a large oilfield identified in this area, and the main reservoir is the carbonate formation. In preliminary explorations, the test production was far lower than expected, indicating serious reservoir formation damage during drilling and completion operations. The damage of such reservoir formations can be generated by multiple sources [13]. The mechanisms of reservoir damage can be classified into four types: mechanical; chemical; biological; and thermal processes [14,15,16]. Studies and explorations have indicated that the subsalt carbonate formations in the Middle East have strong heterogeneity. Moreover, micro-fractures, such as oil and gas storage space and seepage channels, develop in some layers [17], which can easily result in serious reservoir formation damage during drilling and completion, leading to productivity far below expectations. In particular, because of the exposure of formations to drilling fluids, formation damage induced by drilling and completion fluids is likely to occur. The invaded filtrates of water-based drilling fluids can cause hydration and swelling of clays in the reservoir formation, reducing the oil-flow passage, and even causing blockage [13,14]. Some surfactants in the drilling and completion fluids can cause wettability alterations of reservoir rock, from hydrophilic to oleophilic, affecting the distribution and flow of oils [18]. Moreover, water-blocking damage may occur, due to the capillary effect of micro-pores and micro-fractures in carbonate formations [13]. In addition, drilling fluids are usually alkaline, and an alkaline fluid with pH exceeding a critical pH may lead to the release of in situ fines, resulting in pore plugging [19]. Therefore, the mechanism of carbonate reservoir formation damage is complex, and needs to be well-investigated before the drilling operations. Radwan [20] proposed a formation-damage-diagnosis workflow, to help diagnose formation-damage problems, and applied it in the El-Morgan oil field. The workflow was used to analyze the potential formation damage of the MS Oilfield in this study.

In order to effectively clean the borehole during drilling in the reservoir formation, high-molecular-weight polymers are required in drill-in fluids, including xanthan gum, polyanionic cellulose, and polyacrylamide [21,22,23,24]. In conventional polymeric drill-in fluids, these polymers, together with filtrate reducers, form a filter cake on the wellbore surface, to mitigate the fluid invasion. As a result, the reservoir formation permeability will also be reduced; it is necessary, therefore, to use gel-breaker solution to destroy and remove the filter cake after drilling operations [25,26,27]. Most commonly used gel breakers are acidic or strong oxidizing substances, which increase the operating costs and present safety risks [28,29,30]. Therefore, it was necessary to study a polymeric drill-in fluid that did not require gel-breaker treatment, in order to improve the safety and efficiency of drilling and completion operations.

**Figure 1:**
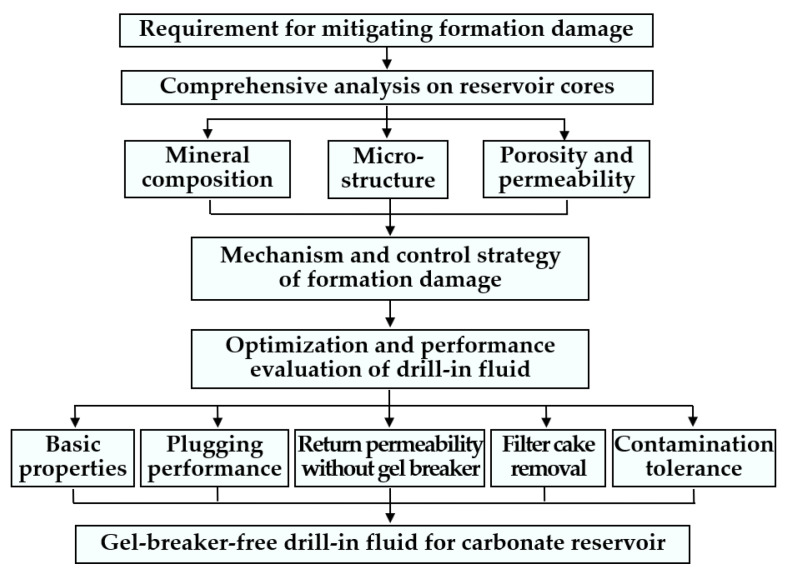
Workflow of this study.

This study aimed to systematically analyze reservoir characteristics and formation-damage mechanisms, and to optimize a low-damage drill-in fluid to mitigate formation damage in drilling and completion operations, thus helping to fully release the productivity in the subsequent production operations. Figure 1 shows the workflow of this study.

## 2. Geological Setting and Reservoir Characteristics

**Figure 2:**
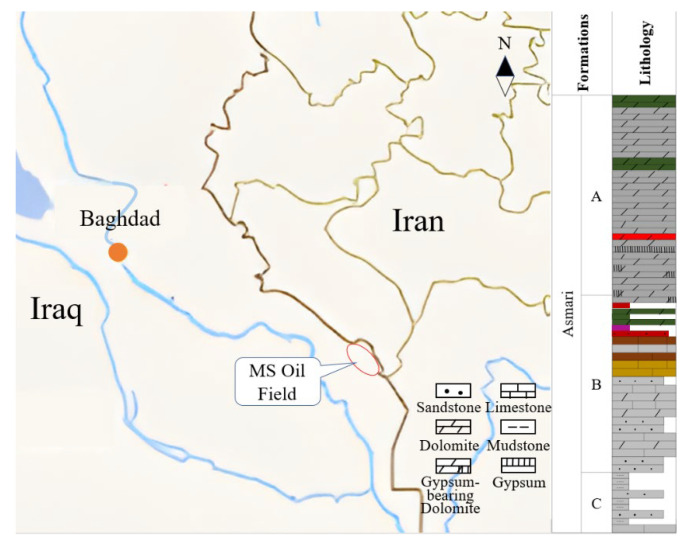
Location and Stratigraphic column of the MS oil field (The Asmali formation is divided into several sections, and the main oil-producing layers are sections A–C).

Figure 2 shows the location and Stratigraphic column of the MS Oilfield [31]. The oilfield is about 350 km away from Baghdad. The Asmari formation is one of the main reservoirs, and the depth is about 2930–3100 m. The formation is mainly limestone, dolomite, sandstone, and mudstone, and the heterogeneity is very strong. The pressure coefficient is 1.16–1.19, and the reservoir temperature is 110–120 °C. It is characterized as a low-porosity and low-permeability reservoir, because the average porosity of the different layers is between 7.1% and 8.7%, and the permeability is between 1.1 and 3.2 mD.

## 3. Results and Discussion

### 3.1. Formation Damage Mechanism Analysis

Firstly, the mineral composition was analyzed. The results for the mineral composition of reservoir core samples at different depths are shown in Table 1. The reservoir rocks of the oilfield were mainly composed of dolomite (87–93%, average 90.25%), followed by anhydrite (3–8%, average 5.5%), with a very small amount of clay minerals (1.25%) and quartz (average 1%), etc. Because the drilling fluid is usually alkaline, dolomite may react with alkali and produce precipitation, which causes pore throat blockage.

**Figure 3:**
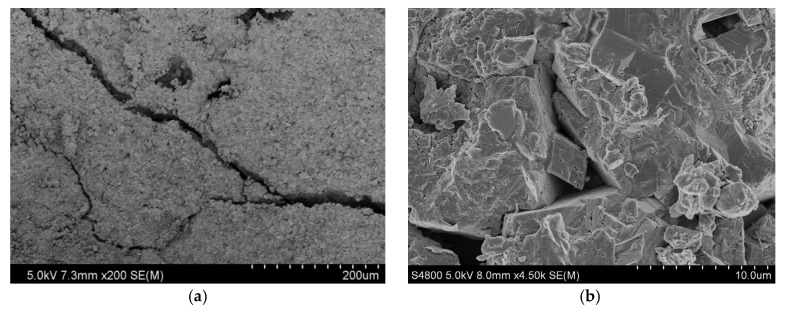

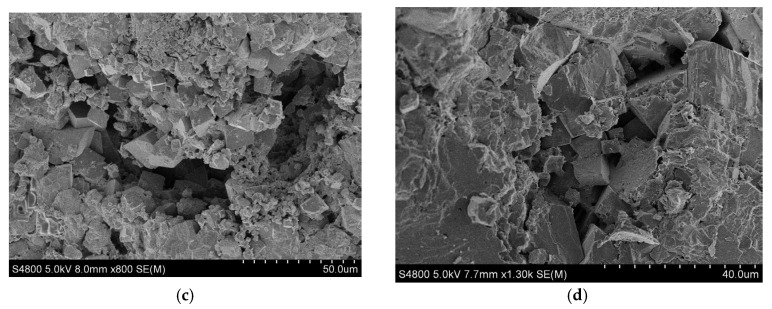
SEM images of reservoir core samples: (**a**) 2991.5 m (200× magnification); (**b**) 3008.9 m (450× magnification); (**c**) 3026.8 m (800× magnification); (**d**) 3029.49 m (1300× magnification).

**Figure 4:**
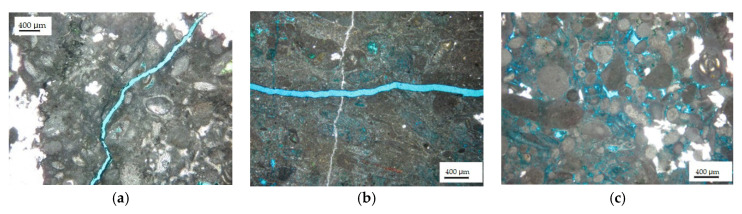
Images of thin-section identification: (**a**) 2982.4 m; (**b**) 2985.8 m; (**c**) 3006.6 m.

Secondly, the microstructure of the reservoir rocks was observed. SEM photographs of reservoir rock samples are shown in Figure 3. Figure 3a shows that there were micro-fractures with a depth of 2991.5 m and a width between 6.7 and 17.2 μm; Figure 3b shows that there were dissolution pores in the formation; Figure 3c,d show that the grain cementation of the rock sample was relatively tight, with the development of intergranular pores, dissolution pores, and micro-fractures, and that there were many interstitial materials, mainly cements. The results of the thin-section analysis of the reservoir rock samples are shown in Figure 4. Figure 4a shows that the fracture width of the core, with a depth of 2982.4 m, was about 80 μm, and that the pore types were mainly intercrystalline pore and intercrystalline dissolved pore with poor connectivity. Figure 4b shows that the fracture width of the core, with a depth of 2985.8 m, reached 120 μm; anhydrite was developed; the pore types were mainly intercrystalline pores and intercrystalline dissolved pores with uneven distribution and poor connectivity. For the core at 3006.6 m, the pores were generally developed, and were mainly intercrystalline pores and intragranular dissolution pores, with uniform distribution and good connectivity; the pore diameter was between 40 μm and 160 μm, as shown in Figure 4c. Therefore, effective temporary plugging of fractures was necessary in the process of drilling, in order to prevent serious reservoir formation damage caused by drilling fluid penetration or even lost circulation [20].

Thirdly, the porosity and permeability characteristics were measured. The results of mercury injection analysis and gas permeability analysis of reservoir rock samples at different depths are shown in Table 2. It can be seen that the reservoir formation porosity and pore throat radius varied greatly at different depths, but that the matrix was relatively tight in general, and that the pore size distribution contributing to the permeability was mainly 1.9–0.16 μm. Therefore, potential water blocking damage might exist [32].

Based on the above experimental results, the mechanism and control strategy of the formation damage was analyzed as follows.

(1) The micro-fractures in the formation provided a natural channel for solids and filtrates of drilling fluids to invade the formation, and even lost circulation of drilling fluids could occur, causing serious formation damage. In accordance with the size of the micro-fractures in the reservoir formation, it was necessary to select a temporary plugging agent that could be subsequently removed, and to reasonably design the particle size gradation, in order to achieve effective temporary plugging of the micro-fractures in the process of drilling in the reservoir formation, thus preventing formation damage caused by drilling-fluid invasion;
(2) Because of the low porosity and permeability of the reservoir formation, the main seepage channels were micro-fractures and micro-throats, which caused high irreducible water saturation of the reservoir rock [13,14]. As a result, it was difficult for oil and gas to flow in the micro-channels, therefore water-blocking damage occurred;
(3) From the point of view of mineral composition, the reservoir rock was mainly composed of dolomite, which could react with alkali liquor to produce a new mineral phase Mg(OH)_2_, as shown in Equation (1) [33]. The mineral particles tended to disperse and migrate to the pore throats, causing a blockage. Accordingly, it was desirable to avoid excessively high pH of the drill-in fluid. Because the content of clay minerals in the reservoir was extremely low (about 1%), there was no problem of potential sensitivity damage caused by clay minerals.

CaMg(CO_3_)_2_ + 2 OH^−^ = Mg(OH)_2_ + CaCO_3_ + CO_3_^2−^ (1)

### 3.2. Drill-In Fluid for Mitigating Carbonate Formation Damage

Firstly, the optimization design of the temporary plugging agent was performed.

In accordance with the ‘ideal filling’ theory and D90 criterion [34], the calculation formula of the maximum particle size of the temporary plugging agent was as follows:

(2) $$D_{\max}=\frac{D_{f}}{{\left(90\%\right)}^{-q}}$$

where *D*_max_ was the maximum particle diameter, *D_f_* was the maximum fracture opening, m; and q was the empirical coefficient, generally 1/3–1/2, and 5/12 in this study.

Based on the Horsefield theory, the optimal particle size of multi-stage close packing could be obtained [35]. This theory assumed that the same particle size was used for packing each time, and that the physicochemical interaction between the particles was ignored. The cumulative mass fraction of particles of each grade could be calculated, and the difference between the particle size values of each grade was the mass fraction of the distribution interval, with the calculation formula being:

(3) $$P\left(D_{n}\right)={\left(\frac{D_{n}}{D_{\max}}\right)}^{q}$$

(4) $$V\left(D_{n}\right)=P\left(D_{n}\right)-P\left(D_{n-1}\right)$$

where *P*(*D_n_*) was the cumulative mass fraction of particles smaller than *D_n_*, %; and *V*(*D_n_*) was the mass fraction of particles in a range of particle diameters *D_n_*_−1_ to *D_n_*, %.

**Figure 5:**
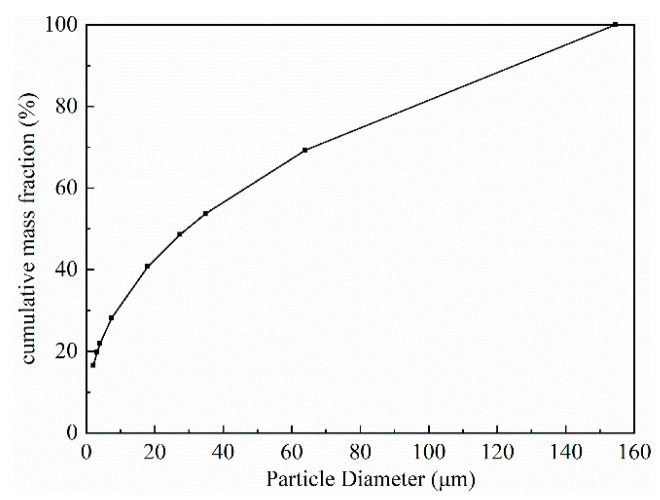
Particle size gradation of the temporary plugging agent.

According to the maximum fracture opening (120 μm) of the reservoir formation, the suitable particle size gradation of the temporary plugging agent could be calculated based on the above equations, as shown in Figure 5. Considering that an acid fracturing stimulation might be used to increase the production in the subsequent operation, an acid-soluble temporary plugging agent was selected, to ensure that it was completely removed in the acidizing process without reducing the productivity.

Based on the control strategy for the formation damage, and considering the comprehensive properties of the drill-in fluid—rheological and filtration properties, lubricity, etc.—the formula of the water-based drill-in fluid was formed: water + 1.6 wt.% FLO (filtrate reducer) + 0.5 wt.% modified xanthan gum (viscosifier) + 0.5 wt.% PAC (filtrate reducer) + 3 wt.% KCl (shale inhibitor) + 6.5 wt.% acid-soluble temporary plugging agent + 1 wt.% JLX (lubricant) + 0.3 HAR (anti-water-blocking agent). The density of the drill-in fluid was adjusted by sodium formate, and the pH value was adjusted by NaOH to 9.5.

The basic properties of the drill-in fluid before and after 16 h hot rolling at 120 °C were tested, and the results are shown in Table 3. The drill-in fluid had good rheological property before (BHR) and after hot rolling (AHR), and the YP/PV ratio was higher than 0.5, which was conducive to suspending and carrying temporary plugging agents and shale cuttings [36]. The API filtrate loss was controlled within 5 mL, indicating its good filtration control performance; the lubricating coefficient was 0.093, and the lubricating property was good.

Because there were shale interlayers and anhydrite in the reservoir formation, the drill-in fluid was required to have good tolerance to Ca^2+^ and shale cuttings; thus, 1 wt.% CaCl_2_ and 8 wt.% bentonite were added to the drill-in fluid, and the rheological property and filtrate loss of the drill-in fluid were tested after hot rolling at 120 °C for 16 h. Table 4 shows that the rheological properties remained stable, and that the fluid loss increased slightly after being contaminated by 1 wt.% CaCl_2_; after being contaminated by 8 wt.% bentonite, the viscosity and yield point increased, but no serious thickening occurred, and the filtration loss remained stable. Therefore, the drill-in fluid had good Ca^2+^ and shale cuttings tolerance performance, and could meet the actual drilling requirements.

**Figure 6:**
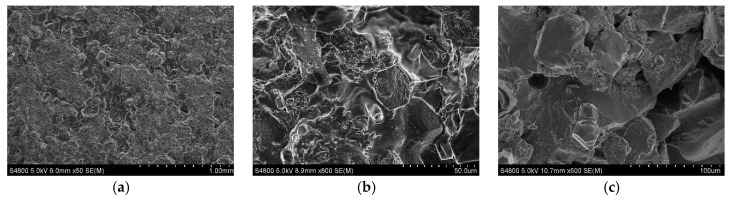
SEM photos of the ceramic disk after the experiment: (**a**) surface (50× magnification); (**b**) surface (800× magnification); (**c**) cross-section (500× magnification).

In plugging performance tests, a ceramic disc with a pore throat diameter of 120 μm was used to simulate the formation fractures, and the plugging performance of the optimized drill-in fluid was evaluated. The results show that, under conditions of 120 °C and 7 MPa, the fluid loss volume was only 4. 8 mL in 30 min. By comparison, the fluid loss of the drill-in fluid designed according to the traditional particle size gradation design criterion was 18.2 mL, and the drill-in fluid without temporary plugging agents was completely lost. After the experiment, the ceramic disk was dried, and observed by SEM (Figure 6); it can be seen that the drill-in fluid formed a tight plugging layer on the surface of the ceramic disk, which effectively reduced the invasion of drill-in fluid into the reservoir formation.

**Figure 7:**
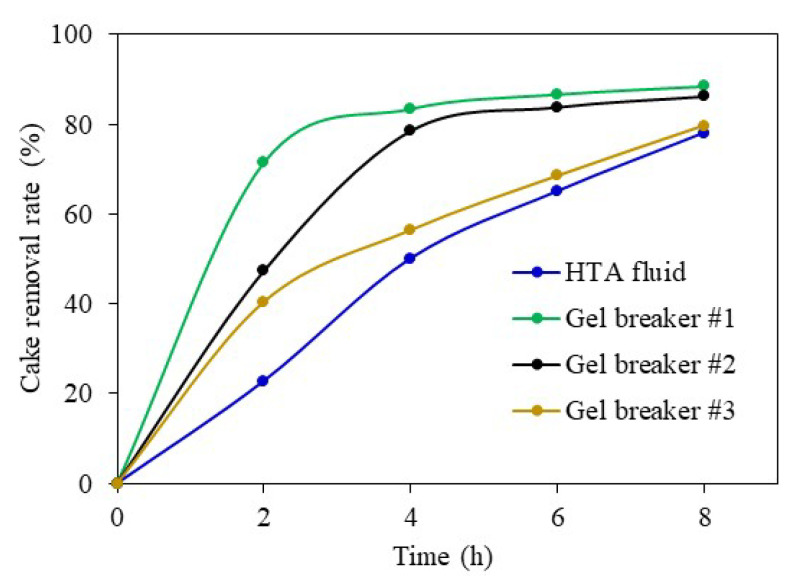
Removal effect of filter cake in different solutions.

The removal effects of the filter cake of the drill-in fluid in the HTA completion fluid, and in the non-acid gel breaker #1, the non-acid gel breaker #2, and the GPC gel-breaker solutions, are shown in Figure 7. The three commercial gel breakers showed obvious cake-removal effects in a short time and, with the increase of soaking time, the removal effects increased. The cake-removal rate reached 88.5% after being soaked in the gel breaker #2 solution. By comparison, the HTA completion fluid did not show a strong gel-breaking effect in the initial state. However, with the increase in soaking time, the removal efficiency of the filter cake increased, and the cake-removal rate reached 78.1% after soaking for 8 h in the HTA completion fluid. The results show that the filter cake of the drill-in fluid can also be effectively removed in the HTA completion fluid without using gel breakers, which is conducive to reducing reservoir formation damage.

The experimental results of the static and dynamic damage experiments with the reservoir formation core samples are shown in Table 5. Under static and dynamic conditions, the return permeability of the core reached 86.35% and 90.67%, respectively, after the end surface of the core sample at the inlet was cut by 0.8 cm. The results indicate that the drill-in fluid can effectively form a temporary plugging layer on the borehole surface, to prevent the filtrate from invading the reservoir formation, and that the temporary plugging layer is thin, which is conducive to the perforation of temporary plugging zones in perforation completion or the removal of temporary plugging zones in acidizing operations. The return permeability values of the core samples which were flooded by drill-in fluid and HTA completion fluid in turn were further tested. The HTA completion fluid consisted of formate brine, 3 wt.% HTA, and 1 wt.% CA101. The results showed that the return permeabilities under static/dynamic conditions were 84.7% and 87.95%, respectively, indicating that the chelating acid completion fluid effectively removed the temporary plugging layer [14,37]. Therefore, a plugging layer formed by the acid-soluble temporary plugging agent, polymer, and starch, can be further removed by acidic completion fluid or subsequent acidification, and the productivity can be fully released.

## 4. Conclusions

In order to efficiently develop the targeted carbonate reservoirs, the formation damage-mechanism was investigated, and a suitable drilling fluid was developed. The conclusions of this study are as follows.

A comprehensive analysis of the reservoir cores showed that the overall porosity and permeability of the carbonate reservoirs in the MS Oilfield in the Middle East were low, and that micro-fractures had developed. The reservoir rocks were mainly composed of dolomite, with a very low content of clay minerals. The main formation damage mechanisms were as follows: the micro-fractures provided channels for the invasion of drilling fluid solids and filtrate; in the formation, there existed potential water-blocking damage; the dolomite reacted with alkaline working fluid, to form precipitation.

Based on the size of the reservoir formation fractures, and the ‘improved ideal filling for temporary plugging’ theory, the particle size distribution of the acid-soluble temporary plugging agent was determined, and a drill-in fluid suitable for carbonate reservoir formation was optimized. The drill-in fluid had good rheological and filtration properties, and could tolerate 1 wt.% CaCl_2_ and 8 wt.% bentonite contaminations. The filter cake could be effectively removed from the HTA completion fluid without gel breakers, which was beneficial to improving operating efficiency and safety. The drill-in fluid could form a tight temporary plugging layer on the surface of the ceramic disc, with pore throat size up to 120 μm, thus reducing the invasion of drill-in fluid into the reservoir formation. Static and dynamic damage experiments showed that the drill-in fluid effectively formed a thin temporary plugging layer on the surface of the core samples, which could be removed by acid completion fluid or subsequent acidification, to release the oil productivity.

## 5. Experimental Section

### 5.1. Materials

The reservoir formation rock samples were obtained from the MS Oilfield, and the depth was 2976–3030 m. The additives of the drill-in fluid and completion fluid—which included modified xanthan gum, modified starch (FLO), polyanionic cellulose (PAC-LV), polymeric alcohol (JLX), fluorocarbon surfactant (HAR), acid-soluble temporary plugging agent, sodium formate, chelating acid (HTA), and corrosion inhibitor (CA101)—were supplied by China Oilfield Services (Sanhe, China). The non-acid gel breakers were supplied by Land Co. Ltd (Shenzhen, China).

### 5.2. Methods

The reservoir formation core samples were analyzed using the following methods. Mineral compositions of reservoir rocks were analyzed with an X’Pert Pro MPD X-ray diffractometer (PANalytical B.V., Almelo, The Netherlands) [38]. The microstructures of the rocks were analyzed using an S-4800 scanning electron microscope (Hitachi, Tokyo, Japan) [39]. The radius distribution of the pore throats, and their permeability contribution, were obtained using capillary pressure curves, which were measured by an Autopore IV9500 mercury intrusion meter (Micromeritics, Norcross, Atlanta, GA, USA) [12]. The rheological properties of the drill-in fluid were tested at 25 °C, using a ZNN-D6 rotational viscometer (Qingdao Haitongda Special Instrument Co., Ltd. Qingdao, China), before and after 16-h hot rolling at 120 °C. The apparent viscosity (AV), plastic viscosity (PV), and yield point (YP) were calculated from the 600 and 300 r/min readings of the viscometer [40]. The filtration loss (FL_API_) of the drilling fluid was measured by a ZNS-2A filtration apparatus (Qingdao Haitongda Special Instrument Co., Ltd.) at 25 °C and 0.7 MPa for 30 min. The permeability plugging apparatus was used to evaluate the plugging performance of the drill-in fluid during drilling [41]. Then, 350 mL of drill-in fluid was injected into the cell. The temperature and pressure in the cell were 120 °C and 3.5 MPa, respectively, to simulate the downhole condition. Filtrates that passed through the ceramic disc were collected at 1 min, 5 min, 7.5 min, 15 min, 25 min, and 30 min, and the volume of the filtrate was recorded. After the experiment, the surface morphology of the dried disk was observed by scanning electron microscopy.

The removal efficiency of the filter cakes after gel breaking was tested as follows. The filter cake was obtained by an API filtration test with the drill-in fluid. The filter cake was dried to a constant weight, and the mass of the filter cake was accurately measured. After that, the filter cake was placed in a gel-breaker solution, or a completion fluid at 90 °C, for standing for different times, and the residual filter cake, after soaking, was taken out, dried, and weighed. The ratio of the mass difference of the filter cake before and after soaking, to the mass of the original filter cake, was the removal rate of the filter cake.

According to SY-T/6540-2002, ‘Lab testing method of drilling and completion fluids damaging oil formation’ [42], the damage degree of the drill-in fluid to the reservoir core samples was evaluated through both the static and dynamic damage experiments.

## Figures and Tables

**Table 1:** Mineral composition of the reservoir rocks.

Depth (m)	Mineral Composition (%)						
	Quartz	Plagioclase	Calcite	Dolomite	Halite	Anhydrite	Clay
2991.5	1	1	-	92	-	5	1
3008.9	1	1	-	93	-	3	1
3026.8	1	1	-	89	1	6	2
3029.5	1	1	2	87	-	8	1

**Table 2:** Results of porosity and permeability characteristic tests.

Depth (m)	Porosity (%)	Permeability (mD)	Max Throat Radius (μm)	Average Throat Radius (μm)	Effect Pore Throat Radius (μm)
2976.6	5.8	0.80	0.974	0.220	0.8–0.16
3008.2	7.6	0.24	1.432	0.325	1.9–0.16
3017.2	19.1	56.6	6.085	2.895	6.3–2.5

**Table 3:** Basic properties of the drill-in fluid.

Condition	Apparent Viscosity (mPa·s)	Plastic Viscosity (mPa·s)	Yield Point (Pa)	Gel Strengh (Pa)	FL_API_ mL	pH	Lubricating Coefficient
BHR	40.0	26.0	14.0	3.5/8.0	3.6	10	
AHR	27.5	18.0	9.5	2.5/4.5	4.9	9.5	0.093

**Table 4:** Effect of CaCl_2_ and bentonite on the properties of the drill-in fluid.

Addition	Condition	Apparent Viscosity (mPa·s)	Plastic Viscosity (mPa·s)	Yield Point (Pa)	Gel Strengh (Pa)	FL_API_ mL
None	BHR	40.0	26.0	14.0	3.5/8.0	3.6
	AHR	27.5	18.0	9.5	2.5/4.5	4.9
1 wt.% CaCl_2_	BHR	41.5	27.0	14.5	3.5/14.0	4.5
	AHR	34.5	24.0	10.5	3.0/5.0	5.6
8 wt.% bentonite	BHR	49.5	32.0	17.5	5.0/9.0	4.0
	AHR	37.0	24.0	13.0	3.5/5.0	5.1

**Table 5:** Results of static and dynamic damage experiments.

Condition	Initial Permeability (10^−3^ µm^2^)	Final Permeability (10^−3^ µm^2^)	Return Permeability (%)	Plugging Removal Method
Static	3.245	2.802	86.35	Cut off the plugging layer
	4.672	3.957	84.7	Completion fluid
Dynamic	3.751	3.401	90.67	Cut off the plugging layer
	5.122	4.505	87.95	Completion fluid

## Data Availability

The data presented in this study are available on request from the corresponding author.
